# Variable-order reference-free variant discovery with the Burrows-Wheeler Transform

**DOI:** 10.1186/s12859-020-03586-3

**Published:** 2020-09-16

**Authors:** Nicola Prezza, Nadia Pisanti, Marinella Sciortino, Giovanna Rosone

**Affiliations:** 1 Dipartimento di Informatica, Università di Pisa, Largo B. Pontecorvo, 3, Pisa, Italy; 2 Dipartimento di Matematica e Informatica, Università di Palermo, Via Archirafi, 34, Palermo, Italy

**Keywords:** SNP, INDEL, BWT, Alignment-free, Assembly-free

## Abstract

**Background:**

In [Prezza et al., AMB 2019], a new reference-free and alignment-free framework for the detection of SNPs was suggested and tested. The framework, based on the Burrows-Wheeler Transform (BWT), significantly improves sensitivity and precision of previous de Bruijn graphs based tools by overcoming several of their limitations, namely: (i) the need to establish a fixed value, usually small, for the order *k*, (ii) the loss of important information such as *k*-mer coverage and adjacency of *k*-mers within the same read, and (iii) bad performance in repeated regions longer than *k* bases. The preliminary tool, however, was able to identify only SNPs and it was too slow and memory consuming due to the use of additional heavy data structures (namely, the Suffix and LCP arrays), besides the BWT.

**Results:**

In this paper, we introduce a new algorithm and the corresponding tool ebwt2InDel that (i) extend the framework of [Prezza et al., AMB 2019] to detect also INDELs, and (ii) implements recent algorithmic findings that allow to perform the whole analysis using just the BWT, thus reducing the working space by one order of magnitude and allowing the analysis of full genomes. Finally, we describe a simple strategy for effectively parallelizing our tool for SNP detection only. On a 24-cores machine, the parallel version of our tool is one order of magnitude faster than the sequential one. The tool ebwt2InDel is available at github.com/nicolaprezza/ebwt2InDel.

**Conclusions:**

Results on a synthetic dataset covered at 30x (Human chromosome 1) show that our tool is indeed able to find up to 83% of the SNPs and 72% of the existing INDELs. These percentages considerably improve the 71% of SNPs and 51% of INDELs found by the state-of-the art tool based on de Bruijn graphs. We furthermore report results on larger (real) Human whole-genome sequencing experiments. Also in these cases, our tool exhibits a much higher sensitivity than the state-of-the art tool.

## Background

Variant calling has become a fundamental task in genomics and metagenomics analyses. With *Variant Calling* is meant the process of identifying variants associated with an individual, or trait, or population, from genomic data. The most typical workflow for variant calling downstream of a genome(s) or exome(s) sequencing, is to map the obtained reads onto a reference genome by means of some alignment tool, and then highlight loci where the reads differ from the reference. Such mapping, however, is time consuming, error prone, and it can even be unfeasible when a reference genome is not available (in this case the analysis should start with an assembly process that reconstructs the genomes before comparing/analysing them, but this is often out of reach in practice for several computational and experimental reasons). In this scenario, there has been a growing interest in *reference-free* (and assembly-free) variant calling methods, that perform their task directly on the raw reads data [1–7]. In the literature one can find reference-free methods and tools for detecting SNPs [1, 4, 7, 8], small INDELs [4, 8, 9], sequencing errors [10–12], rearrangement breakpoints [13] in genomic data, haplotype assembly [14–16], as well as alternative splicing events [2] on RNA-Seq data.

Most of the above mentioned effective tools (e.g. [1–4, 13, 17, 18]) in variant discovery use a de Bruijn graph (dBG), i.e. a directed graph in which the set of nodes corresponds to the set of *k*-mers contained in the reads and two *k*-mers are connected by an edge if (i) they perfectly overlap on *k*−1 nucleotides, and (ii) the corresponding (*k*+1)-mer obtained by concatenating them occurs in the dataset (usually, with a fixed minimum frequency). The identification of variants is carried out by detecting and analyzing *bubbles* in dBG, i.e. pairs of disjoint paths sharing the same source node and target node. The advantages of using dBG include the formalization of several biologically interesting features through specific properties of paths in the graph. The drawback of these dBG representations is the need to set in advance the value of the parameter *k* and the lossy constraint of considering *k*-mers rather than representing the actual whole collection of reads. In particular, coverage information of each *k*-mer, as well as the fact that two or more *k*-mers might belong to the same read (and are thus adjacent in the genome), are important pieces of information that are not usually stored in the de Bruijn graph. The limitation of fixing *k* is also present in [19, 20] where *k*-mers are indexed, and in the methods of [21, 22] that use a *Gk array*, while other methods that use suffix array based data structures (like PgSA [23]), instead, do not have a fixed *k*-mer size.

The Burrows-Wheeler Transform (BWT) of a text *T* is a suitable permutation of the letters of *T*, and it has become a fundamental tool for the design of self-indexing data structures. This permutation has been intensively studied from a theoretical and combinatorial viewpoints [24–30] and has found important and successful applications in several areas in science and engineering [7, 20, 31–40], but so far it was not yet used *per se* for direct detection of variants. The eBWT is an extension of the BWT to collections of strings that has been introduced in [41]. In [42], the authors described fast and RAM-efficient methods capable of computing the eBWT of sequence collections of the size encountered in human whole genome sequencing experiments. The eBWT of a read set can be built by using e.g. [43–47]. In [48] the eBWT was used to index reads from the *1000 Genomes Project* [49] in order to support *k*-mer search queries. An eBWT-based compressed index of sets of reads has also been suggested as a basis for both RNA-Seq [50] and metagenomic [51, 52] analyses.

In this paper, we present a new eBWT based strategy for SNPs and INDELs detection in a dataset of “raw reads”. Unlike the usual methods, our tool takes as input the (extended) Burrows-Wheeler Transform of the read collection. The strength of BWT-based indexing for variant calling stems from the facts that the BWT tends to group symbols that share the same right context into *runs* of letters and, remarkably, that the original text can be reconstructed from it (thus allowing the BWT to represent the original string without loss of information), see [24, 26, 53–55]. In [6, 7] we exploited this property and introduced the *Positional Clustering* framework via a series of theoretical results that overall led to the characterization of SNPs as clusters in the BWT of the reads collection, and to detect such clusters using two additional data structures: the *Longest Common Prefix Array* (LCP) and the *Suffix Array* (SA) of the dataset. We thus implemented a SNPs calling tool that we experimentally validated: its accuracy in terms of sensitivity and precision was very promising and competitive, with running time and memory usage comparable when limited to the actual search phase. However, when taking into account the preprocessing phase, and thus the cost of computing LCP array and SA (in addition to the eBWT), the positional clustering based tool for SNPs detection resulted to be overall computationally inefficient. In this work, we overcome the above limitation by using a recent algorithmic result [56] that shows how to extract the LCP*on-the-fly* from the eBWT. Moreover, combinatorial properties of the (LF mapping of the) BWT are used to replace the Suffix Array. As a result, we wrap up a new algorithm that exploits the positional clustering for variant detection using the BWT only, and hence reduces the working space by an order of magnitude. Furthermore, in the resulting tool EBWT2INDEL, we extend the positional clustering algorithmic framework to the detection of INDELs. Experimental results show that our tool exhibits a much higher sensitivity than the state-of-the art tool.

## Methods

### Definitions

Consider a string *s* of length |*s*| from some finite ordered alphabet *Σ*={*c*_1_,*c*_2_,…,*c*_*σ*_} of size *σ*. In this paper, we will work only with the DNA alphabet *Σ*_*DNA*_={*$*,*A*,*C*,*G*,*T*} (augmented with a special end-marker *$*, read below), therefore *σ* will be assumed to be constant with respect to |*s*|. We assume a total order < on alphabet characters such that *$*<*c* for all *c*∈*Σ*∖{*$*}. We denote the characters of a string *s* by *s*[1],*s*[2],…,*s*[|*s*|], where |*s*| is the length of *s*. A *substring* of *s* is denoted as *s*[*i*,*j*]=*s*[*i*]⋯*s*[*j*], with *s*[..*j*]=*s*[1,*j*] being called a *prefix* and *s*[*i*..]=*s*[*i*,|*s*|] a *suffix* of *s*.

Consider now a string *s* of length *n* terminated by the end-marker *$*. If we imagine placing the suffixes of *s* in lexicographic order $s[i_{1}..] < \dots < s[i_{n}..]$, then the Burrows-Wheeler Transform (BWT) of *s* [57] is defined as the unique permutation bwt(*s*) of *s* such that bwt(*s*)[*j*]=*s*[*i*_*j*_−1] if *i*_*j*_>1, and bwt(*s*)[*j*]=*$* otherwise. Each symbol in the BWT is therefore associated with a suffix of the string. Note that, from this definition, the symbols in *s* preceding the same right context *w*∈*Σ*^∗^ (i.e. *w* is a common prefix of the suffixes starting after those symbols) are consecutive in the BWT. Perhaps, the two most important of its many interesting properties are that (i) the BWT is reversible, in the sense that *s* can be reconstructed from *bwt*(*s*) with no additional information [58] and (ii) the clustering effect of the produced output, i.e. the BWT tends to group together symbols that occur in similar contexts in the input string, making the output easy and fast to compress (see, for instance, [53, 59]).

We now consider the generalization of the above notions to a collection $\mathcal {S}=\{R_{1},R_{2},\ldots,R_{m}\}$ of *m* strings (also called *reads* in what follows, due to our target application). We assume that each string is terminated by the end-marker character *$* (common to all strings). In this generalized setting, $n = \sum _{i=1}^{m} |R_{i}|$, i.e. the sum of the lengths of all strings in $\mathcal {S}$. One way to generalize the notion of BWT to such a collection of strings, while also keeping its desirable properties, is to imagine that the end-markers ending the strings are distinct, i.e. that each member *R*_*i*_ of the collection is terminated by a distinct end-marker^1^*$*_*i*_ such that *$*_1_<…<*$*_*m*_<*c*, for any other *c*≠*$*_*i*_ of *Σ*. Then, as described above, we sort the suffixes of all strings in $\mathcal {S}$ and concatenate their preceding characters. The resulting string is called *extended Burrows-Wheeler Transform* (eBWT), see [41, 42]. Another important property of the (e)BWT is the so-called *LF mapping*: the *i*-th occurrence of character *c* on the BWT and the first character of the *i*-th lexicographically-smallest suffix starting with *c* correspond to the same position in the input string (or collection of strings). We indicate with *LF* the function mapping eBWT positions to suffixes (in lexicographic order) as described above, and with *FL* its inverse (FL is also known as the *Ψ* array). The LF mapping stands at the core of the BWT’s reversibility property and of *backward search*, an elegant algorithm used on BWT-based indexes to find the range of suffixes prefixed by a given string. The basic step of backward search consists in finding, given the range of suffixes prefixed by a string *p*, the range of suffixes prefixed *a*·*p*, for any character *a*∈*Σ* (see [58] for more details). On constant-sized alphabets (like our *Σ*_*DNA*_), this step can be implemented in constant time. In this paper we will use backward search to efficiently find a consensus string among the strings preceding a given range of suffixes.

The *longest common prefix* (LCP) array of $\mathcal {S}$ is the array $\textsf {lcp}(\mathcal {S})$ of length *n*, such that $\textsf {lcp}(\mathcal {S})[i]$, with 2≤*i*≤*n*, is the length of the longest common prefix between the *i*-th and (*i*−1)-th lexicographically-smallest suffixes, and $\textsf {lcp}(\mathcal {S})[1] = 0$. If a unique end-marker *$* is used to end strings in $\mathcal {S}$, then end-markers belonging to distinct strings are treated as different characters in the above definition. In what follows, we will simply write lcp[*i*] as a shorthand for $\textsf {lcp}(\mathcal {S})[i]$ when $\mathcal {S}$ will be clear from the context.

The problem we aim at solving in this paper is that of finding variants within a read set without mapping the reads onto the reference genome (alignment-free), and thus without actually needing a reference (reference-free). More formally, given as input the eBWT of a read set $\mathcal S$, our task is to output frequent enough (i.e. covered-enough in the reads dataset) SNPs and INDELs $T_{1} \rightarrow T_{2}$, with $T_{1}, T_{2} \in \Sigma _{DNA}^{*}$ (e.g. *T*_1_=CGT and *T*_2_=C means that the INDEL $\text {\texttt {CGT}} \rightarrow \text {\texttt {C}}$ is present in the dataset) surrounded by a user-defined number of bases (in the example above, we may output the pair $(\mathtt {AT\underline {CGT}CT},\mathtt {AT\underline C CT})$, where the INDEL is underlined). Such procedure can be interpreted as a filtering process that isolates statistically-significant fragments containing variants (note that the output is considerably smaller than the input). This could be useful, e.g. as a pre-processing step to speed up subsequent alignment against a database containing known variants, or simply as a direct quantification of the number of variants in the input dataset (the output’s size is proportional to this number).

### Positional clustering

A crucial property of the eBWT is that the symbols preceding suffixes that begin with some context *w* (a suitable *w* will be defined later) will result in a contiguous substring of $\textsf {ebwt}(\mathcal {S})$. We call this substring a *cluster*. A similar concept has been used in [60] for the reference-free compression of sequence quality scores and in [52] for an alignment-free and assembly-free framework for metagenomic classification. The general idea is that if *all* symbols in a cluster associated to *w* are equal to a symbol *c*, then every occurrence of *w* in $\mathcal {S}$ is preceded by *c*. The opposite situation is more interesting for our purposes: if a cluster contains at least two distinct symbols and *w* is long enough, then those symbols may reflect a variant (e.g. a SNP or the right end of an INDEL) in the input dataset. We call *positional clustering* this variants characterization on the eBWT.

Based on the above observation, in this paper we describe a reference-free and alignment-free tool able to find SNPs and INDELs in an input read set by analyzing just its eBWT. More precisely, the key observation at the core of our tool is that, since variants share the same right-context, they are clustered when we suffix-sort all the reads’ suffixes. As a consequence, the string $\textsf {ebwt}(\mathcal {S})$ can be partitioned in clusters (substrings), each containing the sequenced copies of a fixed position in the underlying (unknown) genome. If that position contains a variant in the read set (e.g. a bi-allelic site, a SNP, or an INDEL), then the cluster will contain more than one distinct symbol. The properties of eBWT clusters were studied in [6], where the following definition was given: clusters are maximal substrings ebwt[*i*,*j*] such that the suffixes in the lexicographic range [*i*,*j*] are prefixed by a string (context) *w* that appears only once in the genome. In particular, *w* is chosen to be the shortest such prefix. When such a cluster can be defined (i.e. *w* does not fall in a repetitive region; note that this event is less frequent with longer reads), this definition guarantees that characters in ebwt[*i*,*j*] are the sequenced copies of a single genome position. At this point, one question arises naturally: given that the underlying genome is unknown, how can we compute the contexts *w* (and thus the clusters)? The following theorem, proven in [6], answers the question: with high probability (dependent on the sequencing error rate), clusters correspond to maximal intervals ebwt[*i*,*j*] that do not contain local LCP minima, i.e. no index 1<*i*≤*r*≤*j*<*n* satisfies lcp[*r*−1]≥lcp[*r*]<lcp[*r*+1]. In order to filter out clusters corresponding to short random contexts *w* (which are statistically not significant), also a minimum LCP is required: for some fixed *k*_*min*_ (by default, *k*_*min*_=16), the suffixes in the cluster must share at least *k* characters (i.e. lcp[*r*]≥*k*_*min*_ for *i*≤*r*≤*j*). Note that this characterization does not need the underlying reference genome to be known, and thus allows us to find clusters with a simple linear scan of the LCP array. Importantly, and differently from tools using a context of fixed length (e.g. those based on de Bruijn graphs), the length |*w*| of our contexts is *variable-order* (i.e. dependent on the particular cluster, thus data driven and not fixed a priori), and can be up to the full read length for high-enough coverages and small-enough error rates.

With our preliminary tool for SNPs detection, described in [7], we showed that the positional clustering framework is indeed able to considerably improve the sensitivity of state-of-the-art tools based on de Bruijn graphs. This outcome was expected, since those tools (i) throw away important information such as the coverage of each *k*-mer and adjacency of *k*-mers inside the input sequenced fragments, and (ii) are not able to disambiguate repetitive regions longer than *k* (even if *k* could be much smaller than the read length). One important drawback of our tool, however, was the need to compute the generalized Suffix Array of the collection (required to efficiently extract contexts from the eBWT), as well as its eBWT and LCP array. This resulted in huge memory demandings: letting *n* be the total number of bases in the collection, the input alone took 7*n* bytes of space on disk. In this paper, we employ recent algorithmic findings described in [56] and improve our tool so that it only needs the eBWT as input. Local LCP minima are computed on-the-fly by simulating a suffix tree traversal (using just the eBWT). Furthermore, we no longer need to compute the generalized Suffix Array, as contexts surrounding variants are extracted using the eBWT’s *LF mapping* property. Moreover, we extend our preliminary tool so that also INDELs can be detected. As a result, we are able to process much larger data sets and become competitive with existing alignment-free tools that find SNPs/INDELs within one or more read sets.

### Data structures

In [56], the authors showed that the LCP of a collection $\mathcal {S}$ of total size *n* on alphabet [1,*σ*] can be computed from $\textsf {ebwt}(\mathcal {S})$ in $O(n\log \sigma)$ time using $O(n\log \sigma)$ bits of working space on top of the input and output size. The algorithm works by simulating a visit of the generalized suffix tree of the collection using just its eBWT. For each visited suffix tree node *x*, the algorithm induces the LCP values whose coordinates stand between the eBWT ranges of the children of *x*. At the end of the visit, all LCP values have been filled. Describing this algorithm is out of the scope of this article; the interested reader can find all the algorithmic details in [56].

We note that, in order to implement the positional clustering strategy, we do not require the whole LCP array: we just need to know which of its entries are larger than *k*_*min*_ and which are local minima, i.e. our goal is to compute two bitvectors *K*_*min*_[1..*n*] and localMin[1..*n*] defined as *K*_*min*_[*i*]=1 if and only if lcp[*i*]≥*k*_*min*_ and localMin[*i*]=1 if and only if lcp[*i*−1]≥lcp[*i*]<lcp[*i*+1]. Similarly, for a fixed parameter *k*_*right*_ (by default, *k*_*right*_=30) we compute an additional bitvector *K*_*right*_[1..*n*] defined as *K*_*right*_[*i*]=1 if and only if lcp[*i*]≥*k*_*right*_. This bitvector will be used to know from which suffix to extract the *k*_*right*_ characters that will form the right-context of our output events (for more details, read the next section). It is easy to modify the algorithm of [56] to compute these three bitvectors instead of the LCP array, and we do not describe the technical details here. By using the same data structures described in [56] (a cache-efficient packed string on alphabet *Σ*_*DNA*_), the total space used in RAM by our variant calling algorithm (which we will describe in the next subsection) is just 7*n* bits (including the bitvectors *K*_*min*_,*K*_*right*_, and localMin), i.e. less than the ASCII-encoded input dataset which, stored in fasta format, takes at least 8*n* bits of space on disk. This space doubles if the user wishes to include also the reverse-complemented dataset, a recommended operation that considerably improves the sensitivity and precision of the method (see [6, 7]).

### Variant calling algorithm

**Fig. 1:**
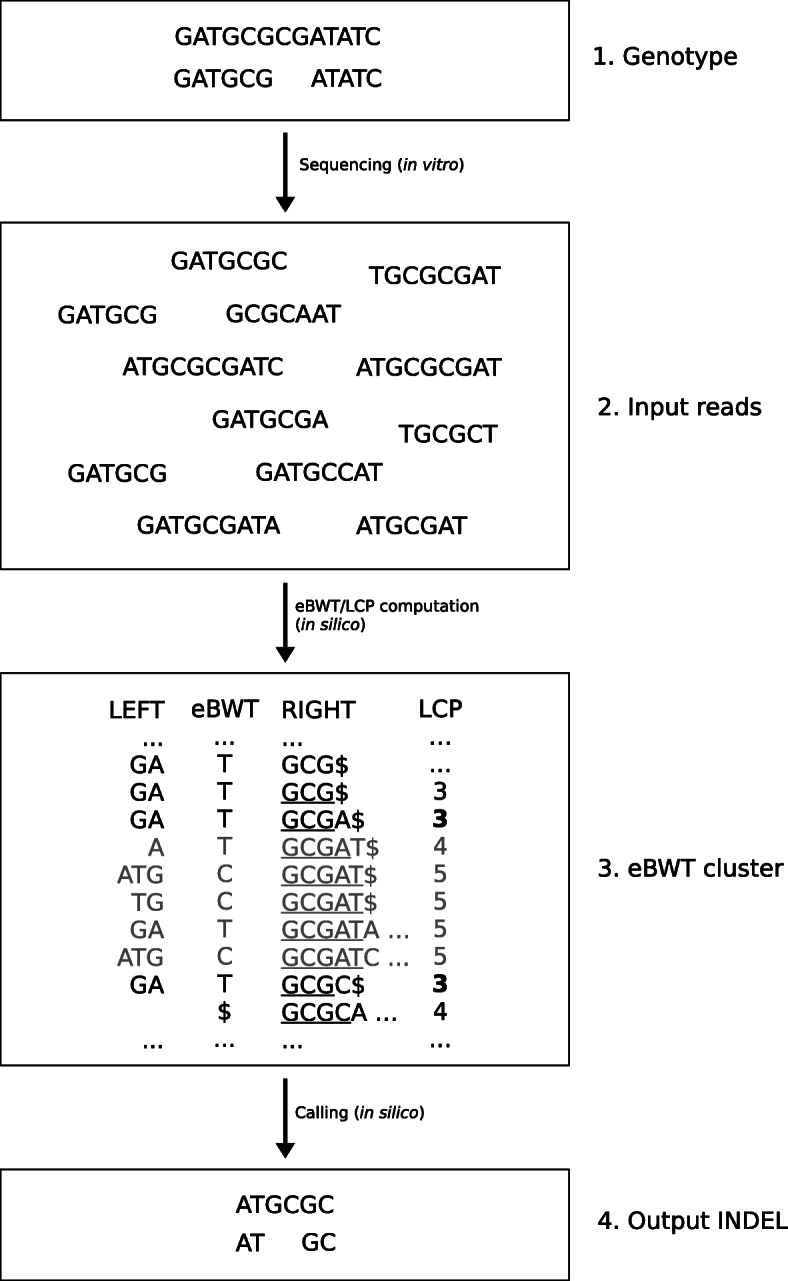
Strategy for finding SNPs/INDELs. **1** Underlyng (unknown) genotype, including an INDEL. **2** Input reads sequenced from the genotype (including sequencing errors). **3** eBWT, LCP, and contexts preceding (LEFT) and following (RIGHT) each eBWT character. In bold: LCP minima. In gray: eBWT cluster. Note that we explicitly compute only column eBWT (the other columns are shown only for illustrative purposes). LCP minima are computed on-the-fly, whereas contexts LEFT and RIGHT are reconstructed using backward search and the FL mapping, respectively. **4** Output INDEL $\mathtt {TGC \rightarrow T}$, extended by one nucleotide to the left and two to the right. Note that the output INDEL is left-shifted, whereas originally (in the unknown genotype) it was right-shifted. To call the INDEL, we (i) compute (via backward search) the two consensus sequences AT and ATGC of the two alleles’ left-contexts (i.e. the strings obtained by concatenating symbols in LEFT and eBWT), and (ii) align them, possibly allowing an INDEL to their right-end. In the figure, the best alignment is the one that deletes GC from ATGC. SNPs are computed similarly, the only difference being that the best alignment of the left-contexts does not introduce insertions nor deletions

The eBWT and arrays *K*_*min*_,*K*_*right*_, and localMin are all we need to find variants (i.e. SNPs and INDELs) in the input dataset. Figure 1 illustrates the process of finding an INDEL (SNPs are found similarly, read below). As discussed above, an eBWT cluster is a maximal substring ebwt[*i*,*j*] such that *K*_*min*_[*r*]=1 and localMin[*r*]=0 for all *i*≤*r*≤*j*. For each eBWT cluster ebwt[*i*,*j*] (in Fig. 1, the cluster is highlighted in gray: TCCTC), we proceed as follows. (i) If the cluster does not contain at least two distinct letters, each occurring at least *min*_*cov*_ times (by default, *min*_*cov*_=6; this is a parameter that can be modified using option -m), then we discard it. Otherwise, we proceed with the next step. (ii) For each pair (*c*_1_,*c*_2_) of distinct letters occurring at least *min*_*cov*_ times in the cluster we do the following:

- We compute a consensus *L*_*t*_, for values *t*=1,2, of length at most *M*=*k*_*left*_+*max*_*indel*_ – where by default *k*_*left*_=31 (this is a parameter that can be modified using option -L) and *max*_*indel*_=10 (this is a parameter that can be modified using option -g) – among suffixes of strings preceding and including character *c*_*t*_ in the eBWT (in Fig. 1, the strings LEFT [*r*]·ebwt[*r*], for *i*≤*r*≤*j*). The consensus – which is defined to be the most frequent string preceding the variant – is computed using backward search: let ebwt[*l*_1_,*r*_1_] be the range obtained by left-extending ebwt[*i*,*j*] with letter *c*_*t*_. Starting from ebwt[*l*_1_,*r*_1_], for *M*−1 times we extend the current eBWT range ebwt[*l*_*q*_,*r*_*q*_] ($q=1,\dots, M-1$) with the most frequent letter in ebwt[*l*_*q*_,*r*_*q*_] (possibly stopping earlier if the range becomes empty). In the example reported in Fig. 1, we have *c*_1_=T,*c*_2_=C and, by choosing *k*_*left*_=2 and *max*_*indel*_=2, we obtain *L*_1_=AT and *L*_2_=ATGC.
- Using function FL, we extract a prefix *R* of *k*_*right*_ characters from the *r*-th smallest suffix for an arbitrary index *i*≤*r*≤*j* such that *K*_*right*_[*r*]=1, where by default *k*_*right*_=30 (this is a parameter that can be modified using option -R). In Fig. 1, we choose *k*_*right*_=2; string *R* is extracted from array RIGHT (in the figure example, any index *r* in the cluster is valid) and corresponds to GC.
- We find the alignment between *L*_1_ and *L*_2_ that minimizes the variant of edit distance where at most one insertion or deletion is allowed at the right-end of the two strings (and nowhere else). Since we know where the INDEL ends, the optimal alignment can be found in linear time. If the optimal alignment does not include INDELs, then the event is a SNP. If the optimal alignment introduces too many edits (by default 2, including the central SNP/INDEL; this is a parameter that can be modified using option -v), then the event is discarded. This is an additional filter that allows discarding left-borders of long repetitive regions, which differ in the contexts preceding the suffixes in the cluster and are therefore captured by the above strategy. In the example of Fig. 1, the optimal alignment is the one that deletes GC from *L*_2_=ATGC.
- We output the sequences *L*_1_·*R* and *L*_2_·*R*, as well as information useful to localize the SNP/INDEL (i.e. length and position in the two fragments), and the coverage of the two sequences in the input dataset.

Note that, by definition of our procedure, INDELs necessarily end with two distinct characters. For example, in Fig. 1 the output INDEL is $\mathtt {TGC \rightarrow T}$. This is due to the fact that those two distinct characters are the ones appearing in the cluster, and we only process clusters containing at least two distinct characters. The effect of this strategy is that, in general, INDELs called from reads aligning on the forward strand will be left-shifted (note: in Fig. 1, the output INDEL is left-shifted, whereas in the original genotype it was right-shifted), whereas INDELs called from reads aligning on the reverse strand will be right-shifted. This is a characteristic shared by several INDELs calling tools since, in principle, it is not possible to decide the correct shift of the INDEL based only on the information contained in the reads. Typical solutions to this ambiguity include using post-processing tools able to normalize INDEL shift in .vcf files (which requires producing a .vcf from our output, read next section), see, e.g. [61].

### VCF creation

If a reference genome is available, then the variants called by our tool can be aligned against it and converted to .vcf format. This can be useful, for example, to compare our output against a database of known variants and, in particular, to validate the output of the tool (see next section). In the next paragraph we describe a pipeline based on the aligner bwa-mem^2^.

First of all, variants can be filtered by minimum coverage using our executable filter_snp. In general, a higher minimum coverage will increase precision and decrease sensitivity (see “Results” section). The next step is to convert our calls to fastq format. This can be achieved, for example, using the tool seqtk^3^ (with the command seqtk seq -F ’I’ in.fa > out.fq) that creates a .fastq file with one entry per output fragment and with dummy high base qualities. Then, the .fastq file can be aligned against the reference genome using bwa-mem. The resulting .sam file can finally be directly converted to .vcf format using our executable sam2vcf. This tool converts every mismatch and INDEL contained in the alignments into a .vcf entry. In our repository we provide a script (snp2vcf.sh) that automatizes this process. Finally, as an optional stage (that was performed in our experiments) one can remove duplicated variations by sorting the .vcf by coordinate and then removing duplicate lines.

### Validation

We used hap.py (https://github.com/Illumina/hap.py) to validate the variations output by the tools. This tool is among the gold standards solutions to compute *sensitivity*$=\frac {TP}{TP+FN}$, *precision*$=\frac {TP}{TP+FP}$, and *F1 score*$=\frac {2TP}{2TP+FP+FN}$ (*TP*,*FP*,*FN* being true positives, false positives, and false negatives, respectively) of a given .vcf against a ground truth .vcf.

### A parallel pipeline for SNP detection

We now describe a simple strategy that allows to effectively parallelize our tool, at the price of limiting the analysis to SNPs only. The idea is to sort the input reads by similarity using the tool HARC^4^[62]. This tool clusters overlapping reads that share a (long) prefix/suffix up to a (small) Hamming distance; as a consequence, the sorted fasta file contains the reads approximately in their order of appearance on the underlying genome. We then break the sorted file into *t* pieces containing approximately the same number of reads. Under the assumption that the reads are sorted by their mapping position on the genome, the *t* pieces can be processed independently (i.e. by building the eBWT and running our tool on each of them). At the end, the union of the *t* outputs (that is, the called variations) will essentially be the same as the one produced by the sequential pipeline. We note that there could be some small differences between the output of the two (sequential and parallel) pipelines due to the fact that, on positions bordering the split points, the coverage is distributed between two fasta’s adjacent pieces. However, in practical applications *t* will be small (i.e. corresponding to the number of processors or servers), therefore we simply ignore this phenomenon in our heuristic. The most important source of noise in this strategy, instead, is that the reads’ order generated by the sorting tool (in this case, HARC) is only an *approximation* of their relative mapping position on the genome. In particular, we note that HARC measures similarity under the Hamming distance, that is, it does not take into account the existence of INDELs among reads. As we show in the next section, this has a strong impact on the sensitivity of INDEL detection of our parallelized tool. Our software repository includes a script, pebwt2InDel.sh, that automatically runs the parallel pipeline (including running HARC, splitting the sorted fasta file, and building the eBWT).

## Results

We compared our EBWT2INDEL with DISCOSNP++ [8], that is an improvement of the DISCOSNP [1, 5] algorithm: while DISCOSNP only detects (both heterozygous and homozygous) *isolated* SNPs from any number of read datasets without a reference genome, DISCOSNP++ detects and ranks all kinds of SNPs as well as small INDELs. As shown in [8], DISCOSNP++ performs better than state-of-the-art methods in terms of both computational performances and quality of the results.

DISCOSNP++ is a pipeline of several independent tools. In the first step, DISCOSNP++ builds the dBG of the input datasets taking into account both the size of the *k*-mers and the minimum coverage *c* (DBGH5 module), and presumed erroneous *k*-mers are removed based on their frequency. Then, DISCOSNP++ detects bubbles [17] generated in the dBG by the presence of SNPs (isolated or not) and INDELs, and it outputs a .fasta file containing the variant sequences (KISSNP2 module). A final step (KISSREADS2) maps back the reads from all input read sets on the variant sequences, mainly in order to determine the read coverage per allele and per read set of each variant. This module also computes a rank per variant, indicating whether it exhibits discriminant allele frequencies in the datasets. The last module generates a .vcf of the predicted variants. If no reference genome is provided, this step is simply a change of format from .fasta to .vcf (VCFCREATOR module).

We performed three experiments: (i) on synthetic data, Human chromosome 1, (ii) on real data, Human chromosome 1, and (iii) on whole-genome sequencing real Human data. We added the reverse-complement of the reads to each dataset in order to improve the sensitivity of our tool (note that DISCOSNP++ performs implicitly this step by adding the reverse-complement of the *k*-mers to the de Bruijn graph as well). We validated the results of experiments (i) and (ii) at different coverages to assess the effect of coverage on the tools’ performance. In the simulated experiment (i), the ground truth, represented by a .vcf file, was known with certainty and thus the experiment had the goal to assess the precision and sensitivity of the tools. The experiments on real data allowed us to assess the sensitivity and speed of the tools in real-case scenarios. The goal of experiments (i) and (ii) was to reconstruct the genotype of individual HG00096 (from the *1000 Genomes Project*’s database), Chromosome 1, by detecting heterozygous sites from the raw reads. Precision and sensitivity of the tools were calculated by comparing the ground truth .vcf with the .vcf generated from the tools’ outputs, by using the pipeline described above. In experiment (iii), we analyzed variations contained in the union of the two whole-genome datasets NA12892 and NA12878 (mother and son, respectively). The goal of this experiment was to compare the number of variations reported by the two tools in a more realistic (and large) scenario.

Both tools allow the user to filter out variants covered less than a fixed threshold. In our case, this is done at post-processing time (using the tool filter_snp) by simply discarding low-covered variants from the output of EBWT2INDEL. In the case of DISCOSNP++, this requires re-building the de Bruijn graph with a different value of parameter -c. For both tools, precision is proportional to this threshold, while sensitivity is inversely proportional. In experiments (i) and (ii), we therefore ran both tools varying this threshold in the range [2,26] and selected the value yielding the best average between the F1 scores of INDELs and SNPs. On synthetic data and coverages 10x, 20x, 30x, 40x, 50x, we obtained the best results for EBWT2INDEL with thresholds 3, 3, 4, 4, 6 and for DISCOSNP++ with thresholds 2, 2, 3, 3, 4, respectively. Similar settings were used on real data.

All our experiments have been run on a 24-core machine with Intel(R) Xeon(R) CPU E5-2620 v3 at 2.40 GHz, and with 128 GB of shared memory. The system is Ubuntu 14.04.2 LTS.

### Synthetic experiment (i) - human chromosome 1

**Fig. 2:**
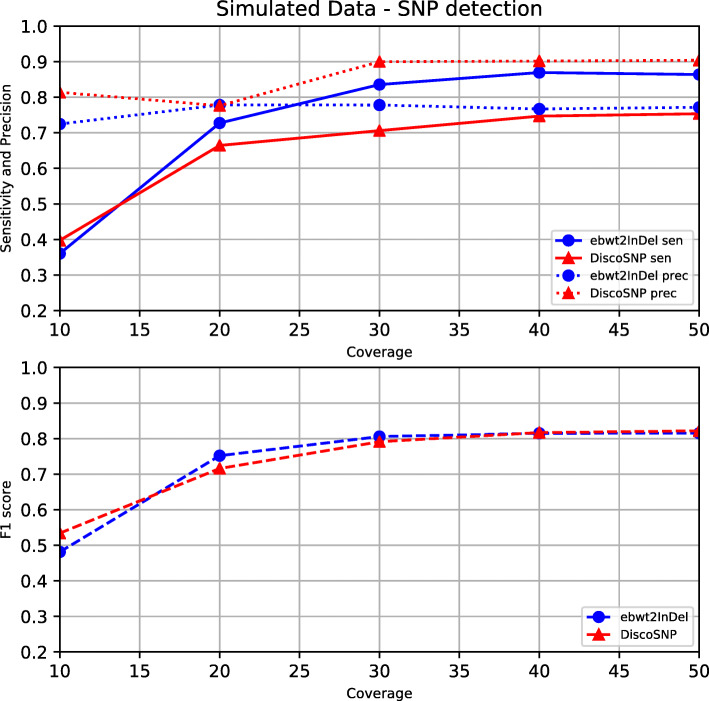
Simulated SNP detection. SNP sensitivity, precision and *F*_1_ score on synthetic data as a function of the dataset’s coverage

**Fig. 3:**
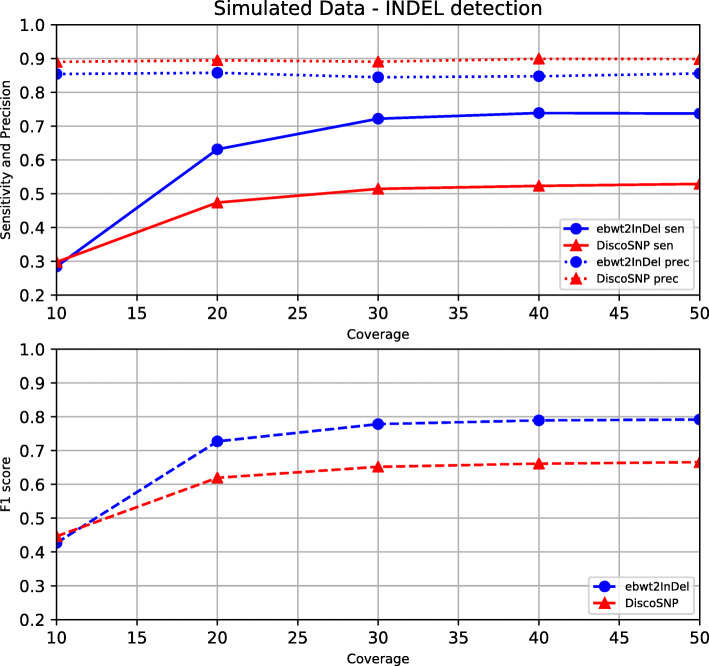
Simulated INDEL detection. INDEL sensitivity, precision and *F*_1_ score on synthetic data as a function of the dataset’s coverage

In the synthetic experiment (i), we generated two variants of human Chromosome 1 by applying to it the two alleles of each heterozygous variation contained in the .vcf file downloaded from ftp://ftp.1000genomes.ebi.ac.uk/vol1/ftp/release/20130502/. The modified bi-allelic chromosome was used to simulate synthetic reads with the tool SimSeq [63], uniformly distributing the coverage among the two chromosome’s variants and using the HiSeq error profile^5^ publicly available in the SimSeq’s repository. We simulated 100-bp synthetic reads with total coverage ranging from 10x to 50x in order to assess the effect of coverage on the sensitivity and precision of the two tools. The results on the synthetic dataset are reported in Figs. 2 and 3.

### Experiment on real data (ii) - human chromosome 1

In the real data experiment (ii) we ran the two tools on a reads dataset (HG00096, Chromosome 1) downloaded from ftp://ftp.1000genomes.ebi.ac.uk/vol1/ftp/phase3/data/. We randomly sampled reads from the original dataset keeping a coverage ranging from 10x to the 48x full-dataset coverage. For the ground truth, we used again the .vcf file used in the synthetic dataset. Unlike in the first experiment, we note that in this case such .vcf was only an approximation of the real ground truth (that is, the individual’s unknown genotype) since it had been created by the *1000 Genomes Project* consortium starting from the raw read dataset that we also used for our experiment. It is therefore expected that the differences between this and the .vcfs created by EBWT2INDEL and DISCOSNP++ are larger with respect to those in the synthetic dataset, with consequent drops in precision for both tools. In Table 1 we report the running times of both tools on the datasets. We separate the running time required to build the BWT (by using BCR [42, 43]) from the running time of EBWT2INDEL for two reasons: first, our tool assumes that the input is represented as a BWT, which is a lossless representation of the input fasta and can therefore replace it; optimizing the BWT-constructions step is a well-studied problem that does not fit the scope of this article. Second, the BWT needs to be built only once for each input dataset. If one wishes to run further analyses on the same dataset, possibly using different parameters, the same BWT can be used. This is not the case for DISCOSNP, whose de Bruijn graph is a lossy representation of the data and depends on the input parameters (*k*-mer length and minimum coverage). In Table 2 we report the sensitivity and precision of the tools on the 30x-covered dataset. We decided to show only the results on this coverage for simplicity of exposition: as in the synthetic experiments, we observed that with coverages larger than 30x the sensitivity and precision of the two tools did not improve significantly.

**Table 1:** Running times on real data

coverage	BCR (BWT)	ebwt2InDel	DiscoSnp++
10	1:03:02	0:51:05	00:54:07
20	2:08:52	1:24:00	01:09:06
30	3:19:18	2:20:14	01:21:31
40	4:22:06	2:55:45	01:37:41
48	5:11:35	3:57:26	01:42:50

We also show the times required to build the BWT using the tool BCR. All tools were run using one core only

**Table 2:** Results on the 30x-covered real dataset

metric	ebwt2InDel	DiscoSnp++
SEN SNP	0.791231	0.641049
PREC SNP	0.596384	0.784806
SEN INDEL	0.547036	0.425699
PREC INDEL	0.533956	0.571847
F1 SNP	0.680127	0.705681
F1 INDEL	0.540417	0.488067

**Comparison with our old tool** ebwt2snp

We also tested the old version of our tool [7] on the 20x real dataset in order to evaluate the improvement in performance of the new version. We recall that the tool [7] needs to compute the Generalized Suffix Array (GSA) of the reads set (this can be achieved using BCR). This (uncompressed) data structure is much larger than the ebwt used by eBWT2CLUST, thus a larger pre-processing time and larger disk usage with respect to eBWT2CLUST are expected. We also note that, once the GSA is computed, the tool [7] only needs to scan this array once to perform the analysis. The post-processing phase therefore is very fast and uses little RAM. On the 20x real dataset, eBWT2CLUST takes 2h 9’ to compute the ebwt (BCR), and 1h 24’ to perform the analysis (ebwt2InDel), using 8.3 GB of RAM and 9.5 GB of disk space (in addition to the input dataset and output). The old tool ebwt2snp requires 14h 13’ to compute the Generalized Suffix Array (BCR) and 28’ to perform the analysis (ebwt2clust + clust2snp) using 1 GB of RAM (it loads fewer structures in RAM) and 73 GB of disk space (in addition to the input dataset and output). Thus, our new pipeline is, overall, much faster and uses one order of magnitude less disk space due to the use of compressed data structures (faster to calculate and lighter to store).

**Enabling Parallelism**

We run EBWT2INDEL and DISCOSNP++ on the 30x-covered real dataset allowing the two tools to use 24 cores, in order to establish the effectiveness of the parallelization strategies of both tools. The parallel pipeline using HARC followed by BCR and EBWT2INDEL (run in parallel on 24 pieces of the sorted fasta file) terminated in 33 minutes. This is approximately 10 times faster than the complete sequential pipeline (see Table 1). By comparing the results with the same ground-truth vcf file used for Table 2, we obtained a SNP sensitivity of 71.68%, a SNP precision of 68.58%, and a F1 score of 70.09% (setting the minimum-coverage parameter -m to 5, which yielded the best sensitivity/precision trade-off). As expected (since HARC works only under Hamming distance), INDEL sensitivity and precision dropped to 5.79% and 30.31%, respectively. DISCOSNP++ terminated the analysis in 28 minutes and achieved the same performance as those shown in Table 2 (the number of cores used by DISCOSNP++ affects only its running times, not its output).

### Real experiment (iii) - whole genome, two individuals

In the first whole genome experiment we reported variations contained in the union of the first 320 million 100-bp reads from both datasets github.com/nicolaprezza/ebwt2InDeland www.internationalgenome.org/data-portal/sample/NA12878 (mother and son, respectively). Note that, first, we have filtered the reads by removing those containing the symbol *N*. In total, the dataset’s size amounted to 65.3 Gbp, that is, a coverage of 10x per individual. We used the same corresponding parameters for the two tools: -m/-c = 3 controls the minimum read/*k*-mer coverage, -g/-D = 10 controls the maximum INDEL length, and -v/-P = 2 controls the maximum number of differences allowed (in the bubble/left-context of clusters) in addition to the main SNP/INDEL. In order to count the number of variations output by the tools, we converted their outputs (that is, .fasta files containing DNA fragment pairs testifying the variations) to .vcf files by aligning them against the hg38 Human assembly (using the pipeline described in “VCF creation” subsection). Finally, we removed duplicate entries from the .vcf files (since both tools analyze the dataset and its reverse-complement, some variants could be found twice: on the forward and reverse strands). In sequential mode, BCR required 23 hours and 19 minutes to build the eBWT, while EBWT2INDEL terminated its execution in 33 hours (the whole pipeline took therefore 56 hours). The process returned 4,265,718 SNPs and 270,488 INDELs. Our parallel pipeline, on the other hand, processed the input in just 8 hours and reported 2,693,867 SNPs and 65,582 INDELs. DISCOSNP++ completed the analysis in about 2 hours and 45 minutes when running in parallel mode with 1947% of CPU utilization, and in 10 hours when using one core only. The pipeline based on DISCOSNP++ returned 787,256 SNPs and 104,090 INDELs.

### Real experiment (iv) - whole genome, one individual

We performed one more experiment whose goal was to find heterozygous sites from a 20x-covered dataset of Human individual NA12878. We selected 1.300.000.000 reads from the file ftp://ftp.sra.ebi.ac.uk/vol1/fastq/SRR622/SRR622457/. The whole dataset, together with the reverse-complemented reads, totalled approximately 122 GBp. For the ground truth, we used the NCBI .vcfftp://ftp-trace.ncbi.nlm.nih.gov/giab/ftp/release/NA12878_HG001/latest/, and validated the calls using hap.py. As in the previous experiment, we used the same corresponding parameters for the two tools: -m/-c = 3, -g/-D = 10, and -v/-P = 2. In order to build a .vcf from the tools’ outputs, we aligned the called variations against the hg38 Human assembly, and removed duplicate entries from the resulting .vcf.

BCR required 21 hours and 24 minutes to build the eBWT, while EBWT2INDEL terminated its execution in 24 hours and 34 minutes (the whole pipeline took therefore 46 hours). The whole pipeline found 55.56% of the INDELs and 85.53% of the SNPs present in the ground truth, with a precision of 47.07% and 35.16%, respectively. DISCOSNP++ completed the analysis in 32 hours and 44 minutes and found 41.68% of the INDELs and 72.18% of the SNPs, with a precision of 51.33% and 63.97%, respectively.

## Discussion

### Synthetic experiment (i) - human chromosome 1

Experiments on synthetic data allowed us to assess how precisely the two tools were able to reconstruct the original .vcf file. The outcome, reported in Figs. 2 and 3, is that EBWT2INDEL finds considerably more SNPs and INDELs than DISCOSNP++, at the price of being slightly less precise. At 10x coverage, most variations are not found by both tools. However, already at 20x the sensitivity of the tools starts to stabilize, reaching stable values at 30x. The plots suggest that increasing the coverage above 30x does not bring significant advantages. At 30x, EBWT2INDEL is able to find nearly all SNPs (93%), and a large fraction of the INDELs (83%). DISCOSNP++, on the other hand, finds only 70% of the SNPs and 50% of the INDELs. The high sensitivity of our tool is moderately paid in terms of precision: at 30x coverage, 77% of the SNPs and 89% of the INDELs output by our tool are correct, versus 90% and 94% of DISCOSNP++. By mixing precision and sensitivity in the F1 metric, we obtain higher scores than DISCOSNP++ across all coverages.

### Real experiment (ii) - human chromosome 1

Also on real data our tool exhibits a much higher sensitivity than DISCOSNP++, especially on INDELs: we are able to find 79.1% of the SNPs contained in the ground truth, as opposed to 64.1% of DISCOSNP++, and 54.7 of the INDELs, as opposed to 42.6% of DISCOSNP++. As expected, both tools exhibit a lower precision with respect to the synthetic experiment, due to the fact that in this case the ground truth was just an approximation of the underlying genotype. By enabling parallelism, EBWT2INDEL’s SNP sensitivity decreases by 7.4%, but precision increases by 8.9%, resulting in an increase of the F1 score by 2%. This is explainable by the fact that, by pre-processing the data with HARC, we separate in different chunks reads that share long sub-sequences (and thus create eBWT clusters) but align on different parts of the genome. As expected, our parallel pipeline finds very few INDELs compared to the sequential one. In a future development of our tool we plan to implement a read-sorting algorithm able to work under edit distance in order to improve this result.

### Real experiment (iii) - whole genome, two individuals

The large processing times of our pipeline are paid off by a much larger number of reported variations: using comparable parameters for both tools, the fragments output by EBWT2INDEL (in sequential mode) contained >5 times more SNPs and >2 times more INDELs than those output by DISCOSNP++. In parallel mode, EBWT2INDEL found less SNPs, though still >3 times more than DISCOSNP++. This depends on the sorting strategy of HARC, which in this case separated reads that, in the sequential pipeline, contained areas that clustered together and also yielded SNPs.

We note that, while the goal of this experiment was just to compare the raw amount of variations found by the two tools in the merged dataset, in a more realistic application one might be interested in discarding heterozygous sites within the same individual and keep only differences across the two individuals. This kind of analysis cannot be performed using DISCOSNP++, since this tool does not “color” the paths of bubbles in the de Bruijn graph according to their provenience from the two input datasets (the *k*-mers of both individuals are pooled together). On the other hand, this analysis can easily be performed with EBWT2INDEL by just comparing the BWTs of the two datasets, or taking as input one single BWT (as we did in our experiment) *and* a bitvector (the so-called *document array*) that tells apart eBWT characters of the two individuals.

### Real experiment (iv) - whole genome, one individual

In this experiment, EBWT2INDEL has been about 50% slower than DISCOSNP++ but found many more variations. As observed in the above real experiments, the precision of both tools is low due to the fact that the ground truth itself is an approximation (and not exact as in the simulated experiments; in this case, a low precision value means that the tool has found more variations than those present in the ground truth.

### Resources

Despite the complex data structures used, the running times of EBWT2INDEL are not much higher than those of DISCOSNP++: using one core for both tools, EBWT2INDEL is 2.3 times slower than DISCOSNP++ on the 48x-covered real dataset, and 1.42 times slower on the 30x-covered real dataset. It is worth to note that, while the running times of our tool scale linearly with the dataset’s size, those of DISCOSNP scale linearly with the dataset’s *complexity* (i.e. number of distinct *k*-mers): this is the reason why the ratio between ours and DISCOSNP++ running times increase with the coverage. If we take into account also BWT construction, this step accounted for 60% of the total processing time in experiments (i) and (ii), and 42% in experiment (iii). By enabling parallelism, our whole pipeline (eBWT construction and SNP analysis) runs in times comparable to those of DISCOSNP++, while also being more sensitive.

As far as the RAM usage of our tool is concerned, we observed that (as expected) it always amounted to 7*n* bits per base; on the largest dataset (50x synthetic), this was equivalent to 21GB. On the (more realistic) 30x real dataset, our tool used 12.7 GB of RAM. The RAM usage of our tool was dominated by the variant calling phase; building the BWT required at most 3GB of RAM on the largest dataset.

## Conclusions

In this work, we described EBWT2INDEL, a new algorithm that detects SNPs and INDELs. We also described a simple strategy for effectively parallelizing our tool for SNP detection only.

We validated EBWT2INDEL on both synthetic and real data. In particular, we simulated synthetic read collections with a range of values for the coverage, to investigate the effect of such parameter on both accuracy and computational performances. In this case the ground truth is known with certainty, and thus the experiment has also the goal to assess the precision of the tools. The experiment on real data aims at assessing the sensitivity and speed of the tools in a real-case scenario. On synthetic 100 bp reads simulated from the Human chromosome 1 at 30x coverage, our tool is able to find 83.5% of the SNPs with an accuracy of 77.8%, and 72.2% of the INDELS with an accuracy of 84.4%. This considerably improves the sensitivity of the state-of-the-art tool based on de Bruijn graphs, who finds 70.6% of the SNPs with an accuracy of 90% and 51.4% of the INDELs with an accuracy of 89%. Similar performance are observed on real data (with lower precision for both tools due to the use of an approximated ground truth). As far as the computational cost of our tool is concerned (excluding eBWT computation), when using only one core we report a slow-down of a factor of 1.4 with respect to the state-of-the-art tool. When including eBWT computation, our whole pipeline is 4 times slower than the state-of-the-art (run on one core only). We then describe a simple way to parallelize our strategy for SNPs detection only. When enabling multi-threading on 24 cores for both tools, our complete pipeline runs in times comparable to those of the state-of-the-art competitor while at the same time also exhibiting a higher SNP precision. As a limitation, our parallel pipeline at the moment works for SNPs only, due to the use of an external pre-processing tool that does not take into account the presence of INDELs in the read set. We plan to overcome this limitation in a forthcoming update of our tool. We furthermore report results on a larger (real) whole-genome sequencing experiment whose input consisted of two 10x-covered Human datasets from the *1000 Genomes Project*. Also in this case, our tool exhibits a much higher sensitivity than the state-of-the art tool, finding >5 times more SNPs and >2 times more INDELs.

## Data Availability

The tool ebwt2InDel is freely available for academic use at github.com/nicolaprezza/ebwt2InDel. Information to generate the simulated datasets is available in the Datasets directory of the same Github repository. The datasets used and/or analysed during the current study are available from the corresponding author on reasonable request.

## Abbreviations

BWT: Burrows-Wheeler Transform
eBWT: Extended Burrows-Wheeler Transform
GSA: Generalized suffix array
LCP: Longest common prefix
SA: Suffix array
dBG: de Bruijn graph
VCF: Variant call format
INDEL: INsertions and/or DELetions
SNP: Single nucleotide polymorphism
FN: False negatives
FP: False positives
TP: True positives

## Footnotes

1 the end-markers are not actually distinct: they are treated as such only inside the sorting algorithm.

2 https://github.com/lh3/bwa.git

3 https://github.com/lh3/seqtk.git

4 https://github.com/shubhamchandak94/HARC

5 https://github.com/jstjohn/SimSeq/blob/master/examples/
